# Coordination of leaf hydraulic and economic traits in *Cinnamomum camphora* under impervious pavement

**DOI:** 10.1186/s12870-022-03740-4

**Published:** 2022-07-16

**Authors:** Cheng Zhang, Huihui Liu, Nuo Huang, Fengyu Zhang, Yanqiong Meng, Jianan Wang, Yiyong Li

**Affiliations:** 1 School of Forestry and Landscape Architecture, Anhui Agricultural University, Changjiang West Road 130, Shushan District, Hefei, 230036 China; 2 Hefei Urban Ecosystem Research Station, National Forestry and Grassland Administration, Changjiang West Road 130, Shushan District, Hefei, 230036 China

**Keywords:** Urban impervious pavement, Leaf economic spectrum, Drought resistance, Adaptation strategy

## Abstract

**Background:**

Paved urban environments can pose great threats to the physiological functioning and ecological services of street trees. In this context, assessment of leaf phenotypic plasticity is crucial for understanding the ecological strategy of tree species under impervious pavements.

**Results:**

In this study, we measured a set of leaf economic traits, hydraulic traits of *Cinnamomum camphora*, and surrounding environmental factors in a street site (the soil was covered by the impervious pavement) and a park site (the soil was covered by grass) in Hefei, eastern China. Compared with the park site, trees in the street site had higher stomatal length (SL), leaf thickness (LT), maximum photochemical quantum yield of photosystem II (Y(II)), and lower stomatal density (SD), specific leaf area (SLA), the leaf water potential at 50% loss of hydraulic conductance (P_50_), and leaf turgor loss point (TLP). Redundancy analysis showed that air relative humidity and volumetric soil water content caused these traits to be altered.

**Conclusions:**

Our results showed that *C. camphora* adapted to the street pavement environment through the coordination of leaf economic and leaf hydraulic traits, and adopted the slow investment return type in the leaf economic spectrum and high drought resistance to meet its actual physiological needs. This finding provides a new perspective for understanding the physiological strategies of street trees to adapt to urban pavement environments.

**Supplementary Information:**

The online version contains supplementary material available at 10.1186/s12870-022-03740-4.

## Introduction

Street trees play an essential role in urban green infrastructure, and they have various potential benefits, including enhancing human health, supporting urban communities, and improving microclimate [1–3]. However, urbanization causes vegetated landscapes to be replaced by concrete and asphalt pavements [4, 5]. Impervious pavement can change climatic conditions such as temperature, soil moisture, and stormwater flow [6, 7]. For example, these surfaces are barriers to water infiltration, which reduces soil moisture. And some pavements with low reflectivity will increase the surface temperature by absorbing more shortwave radiation [8, 9]. These environmental stresses caused by pavement can adversely affect the development of street trees, including changing phenology [10, 11], inhibiting tree growth [12], reducing plant photosynthesis and transpiration [13, 14], and increasing the risk of mortality [15, 16]. Therefore, the study of the tree physiological functions under urban pavements is crucial to urban ecosystem services that support human well-being.

Plant functional traits often reflect the plant’s adaptation to environmental changes [17]. Leaf is one of the critical organs of plants, and its functional traits are a tool for analyzing how plants survive and adapt to the environment [18, 19]. The mechanism explanations of growth and other aspects of the plant in the pavement environment are largely unclear because previous research lacks studies on plant leaf functional traits. Therefore, it is necessary to understand the response and adaptation mechanism of trees to the street pavement environment at the leaf level. Among various leaf traits, leaf economic and hydraulic traits have recently attracted the most attention because of their joint involvement in gas exchange and water conduction in plants [20–22]. One group of traits, known as leaf economic traits, indicates trade-offs between investment and return on carbon and water resources, such as leaf maximum photosynthetic capacity, specific leaf area, and leaf thickness [23, 24]. Another group of leaf traits form leaf hydraulic traits, such as leaf hydraulic conductivity and leaf turgor loss point, which are related to plant hydraulic efficiency and safety under environmental stresses. Recent studies have demonstrated the presence of hydraulic degradation and leaf economic trait trade-offs for trees in urban pavement environments [16, 25].

As leaf functional traits are vital characteristics of plant function, the relationship of leaf functional traits plays an essential role in our understanding of plant physiology, function, and ecological strategies [26, 27]. Because stomata link gas exchange and water loss, leaf economic and hydraulic traits can be coupled on a physiological scale [28, 29]. However, the physical separation of leaf structures and the additional mechanical functions of hydraulic structures may lead to the decoupling of hydraulic and economic traits [30, 31]. Yin et al. found that water availability may be a decisive factor for the relationship between leaf economic and hydraulic traits [28]. Therefore, leaf economic and hydraulic traits may be coordinated to adapt in street pavement drought environments.

*Cinnamomum camphora* is an evergreen broad-leaved tree species widely distributed in the southern part of the Yangtze River Basin in China. This tree species is not only a typical green tree species in China, but also a well-known fast-growing afforestation tree species in other parts of the world [32]. *C. camphora* often dominate subtropical forests in China, so they play an important role in the ecological benefits of subtropical forest ecosystems. Camphor and camphor oil can be extracted from wood and roots, branches and leaves, which are used in medicine and perfume industry [33]. Therefore, it is of great theoretical and practical significance to understand how leaf hydraulic and economic traits of *C. camphora* change in the street environment. The main objectives of this study were to 1) correlate and explain the leaf functional traits with the environmental factors of street pavement; 2) explore the relationships between leaf economic and hydraulic traits in the street pavement environment. We hypothesized that leaf economic and hydraulic traits are coupled in the street pavement environment.

## Material and method

### Study sites and sampling

Experiments were conducted in the urban area of Hefei, the capital of Anhui province, situated in the southeast part of China. The climate of Hefei belongs to the northern subtropical climate zone, an atypical moist and warm subtropical climate. From 2010 to 2020, the construction land area of Hefei increased from 325.91 km^2^ to 466.54 km^2^ [34]. The annual average temperature is 15.7 °C, and the annual precipitation totals 1000 mm [35]. According to the meteorological data from the China Meteorological Data Sharing Service System recorded from 2010 to 2016, the air temperature and rainfall in Hefei are relatively high from June to September (Fig. S1). The species selected for the study is *C. camphora* which has a widespread presence in Hefei and is widely used as a street tree in eastern cities of China.

In September 2021, four individuals per site with good growth were selected as experiment materials. The Yanhe road (117°15′12″E, 31°52′28″N) was selected as the street site. The roadside trees are surrounded by the sidewalk that paves cemented impermeable bricks. The park site was selected as an unpaved control in roomy soil surfaces in the arboretum of Anhui Agricultural University (117°14′36″E, 31°51′45″N). All selected trees were > 10-yr old and have similar diameters. The mean diameters at breast height of selected trees in the street and park sites were 30.05 ± 3.10 cm and 27.30 ± 0.89 cm, respectively. Voucher specimens in the herbarium of School of Forestry and Landscape Architecture, Anhui Agricultural University were identified and deposited by Cheng Zhang.

From 2021 September 2 to September 30 (the record was missing on September 17 due to instrument damage), temperature and moisture in the two sites were measured from 12:00 to 13:00 h. Three individuals were selected from each site. Soil temperature (TS) and soil volumetric water content (SWC) were measured by a data logger (ProCheck, Decagon Devices, USA) with a TEROS 12 sensor inserted into 0–15 cm of soil. The air temperature (TA) and relative humidity (AH) were recorded at a distance of 1.5 m from the ground using a portable weather station (Kestrel 5500, Nielsen-Kellerman, USA).

### Leaf chlorophyll fluorescence

Three fully expanded and mature leaves were collected from the outermost canopy on the south side of one individual plant for the chlorophyll fluorescence measurements (four trees were selected at each site). Therefore, a total of twelve leaves were used for fluorescence measurement from each site.

Data were collected during September 2020 between 20:00 to 22:00 h on nearby leaves to ensure the best possible comparability and accordance of the data sets. And photosynthetic parameters F_v_/F_m_ = F_m_ - F_o_/F_m_ (maximum photochemical quantum yield of photosystem II) and Y(II) = F'm - F′/F'm (photochemical quantum yield of photosystem II) were recorded using a chlorophyll fluorometer (JUNIOR PAM, Walz, Germany). The contribution of PSI fluorescence needs to be considered when citing this data, although it does not affect the comparability of measurements from samples in the two sites [36].

### Plant water status

For each site, leaf water potential, leaf turgor loss point and leaf hydraulic conductivity were measured on four individuals to evaluate plant water status. Fully expanded mature leaves from the sun-exposed top canopy were selected to measure leaf water potential with a pressure chamber (1505D, PMS Instrument Company, USA) in September 2021. Four leaves per individual were collected, immediately sealed in a cool plastic bag and then transported to the laboratory for measurement within 30 min. Predawn leaf water potential (Ψ_pd_) was measured from the leaf in the two sites between 05:00 and 07:00 h, while midday leaf water potential (Ψ_md_) was measured between 12:00 and 14:00 h.

One leafy branch with healthy, mature sun-exposed leaves was collected from each individual and placed in a black plastic bag with damp paper towels, immediately transported to the laboratory under the dark. Each branch was recut in deionized water in the laboratory and rehydrated overnight in a black plastic bag. Six fully expanded and healthy leaves per branch were selected, and the turgor loss point (TLP) was estimated from pressure-volume curves using the bench-drying approach described by Tyree & Hammel [37]. The TLP was obtained from the intersection of the linear and non-linear parts of the pressure-volume curve [38].

According to Brodribb & Holbrook evaporation method in situ [39], 3–5 leaves per individual were used to measure leaf hydraulic conductivity. This technique involved determination of leaf transpiration rate (E), stem water potential (Ψ_x_) and leaf water potential (Ψ_l_). Ψ_x_ was considered equal to the water potential of the covered leaf. Ψ_x_ and Ψ_l_ are measured using the above pressure chamber. Leaf transpiration rate (E) was measured in situ with Licor-6800 photosynthesis system (Licor Biosciences, USA). K_leaf_ was then calculated from Ohms law: K_leaf_ = E / (Ψ_x_ - Ψ_l_).

### Leaf embolism resistance

Leaf embolism resistance was measured in September 2021. Three trees were sampled at each site, usually in the morning and before solar noon, to ensure high leaf water potential. One meter long branches in direct sunlight with about 40 leaves were utilized for the water potential measurements. After the branches were collected, the branch base was cut off another 5 cm in a 5-l plastic bucket with water to avoid arterial embolism and then covered with a black plastic bag. The bagged branches were immediately transported to the laboratory after sampling and hydrated overnight.

The leaves were imaged every 5 min using a custom-built imaging clamp [40, 41]. The imaging area in the center of the leaf was at least 300 mm^2^, covering all venation including the midrib. Image sequence was then analyzed using ImageJ software to determine the embolized pixels for each image [41]. The natural drying time varied from 3 to 4 days, during which the water potential of other leaves (Ψ_leaf_) was measured every 1–3 hours (between 07:00 and 19:00) using a pressure chamber. Taken 2–3 leaves at each time to ensure that Ψ_leaf_ has equilibrated. The laboratory was maintained at 22 °C in the whole process to ensure that the experimental leaf temperature fluctuation was within 1 °C [42, 43]. Leaf xylem vulnerability curves for each individual were obtained by the percentage of accumulated embolized pixels per image to the total embolized pixels of the image sequence and combined with the water potential (Ψ_leaf_).

### Leaf anatomy

On a sunny day after rain in September 2021, four individuals were selected from each site and 2–3 branches were removed from the sun-exposed outer canopy portion of each individual. Then thirty Fully expanded and healthy leaves were collected and stored refrigerated for later anatomical measurement. Leaf area was measured using a BenQ K810 scanner (BenQ, China) and then dried at 65 °C for 48 hours to determine dry weight. From these measurements, the specific leaf area (SLA) was calculated as leaf area / leaf dry weight.

Leaf anatomy measurements were performed on 4–6 leaves per individual, from which 1 cm × 1 cm segments of the central leaf near the main vein were excised and fixed in formalin-acetic acid-ethanol (FAA) fixative for at least 24 hours. Then, leaf fragments were dehydrated using different concentrations of ethanol, embedded in paraffin, sectioned laterally using a rotary microtome (Leica RM2265, Leica Microsystems, Germany), and stained with safranin and fast green. Finally, leaf sections were photographed with a light microscope (Zeiss Primo Star, ZEISS, Germany) at 40× to measure leaf thickness (LT), palisade mesophyll thickness (PT), and spongy mesophyll thickness (ST) [44].

Stomatal density and stomatal length were measured by the blotting method. 15–20 healthy fully expanded leaves were collected from each individual. An even layer of clear nail polish to the middle of the back of the leaf, avoiding the main vein. After 30 minutes of nail polish setting, carefully remove the nail polish film with tweezers and place it on a temporary glass slide. Each slide was then magnified with a light microscope, and five fields of view were randomly selected for image acquisition. Finally, stomatal density (SD) (number of stomata per unit area) and stomatal length (SL) (length between the junctions of the guard cells at both ends of the stomata) were calculated using microscopy software (ZEN blue edition, ZEISS, Germany).

### Statistics

Statistical analysis was performed using R software [45]. Independent t-tests were used to analyze significant differences in environmental factors and values of leaf economic and hydraulic traits between the park and street sites. Leaf xylem vulnerability curves were fitted using a Sigmoidal function using R with the *fitplc* package [46]. The *vegan* package was used for principal component analysis (PCA) of the multivariate association of leaf traits and redundancy analysis (RDA) of assessing the relationship between environmental factors and leaf functional traits [47]. Pearson correlation analysis was used to analyze the internal correlation of leaf functional traits.

## Result

### Environmental conditions

**Fig. 1:**
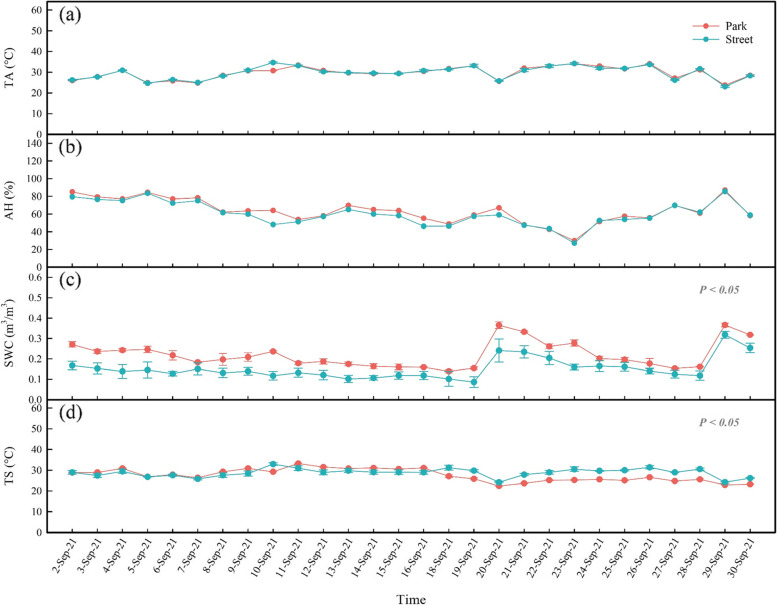
Daily mean values of air temperature (TA; **a**), air relative humidity (AH; **b**), volumetric soil water content (SWC; **c**) and soil temperature (TS; **d**) at study sites park (red) and street (blue) during September 2 to September 30, 2021 (the record was missing on September 17 due to instrument damage). *P* value is calculated using independent t-tests for two sites

It can be seen that AH and TA did not change significantly in the street site (Fig. 1a, b). The mean AH of the street and park sites were 60.4 ± 13.4 and 63.4 ± 13.5, respectively. The mean TA of the street and park sites were 29.8 ± 3.1 and 29.7 ± 3.0, respectively. However, the street pavement environment has a significant influence on TS and SWC. The TS of the street site was 1.23 °C higher than that of the park (*P* < 0.05) (Fig. 1d). The street site had lower SWC than the park site (*P* < 0.05) due to low pavement permeability (Fig. 1c).

### Differences in leaf hydraulic traits between street paved trees and park trees

**Fig. 2:**
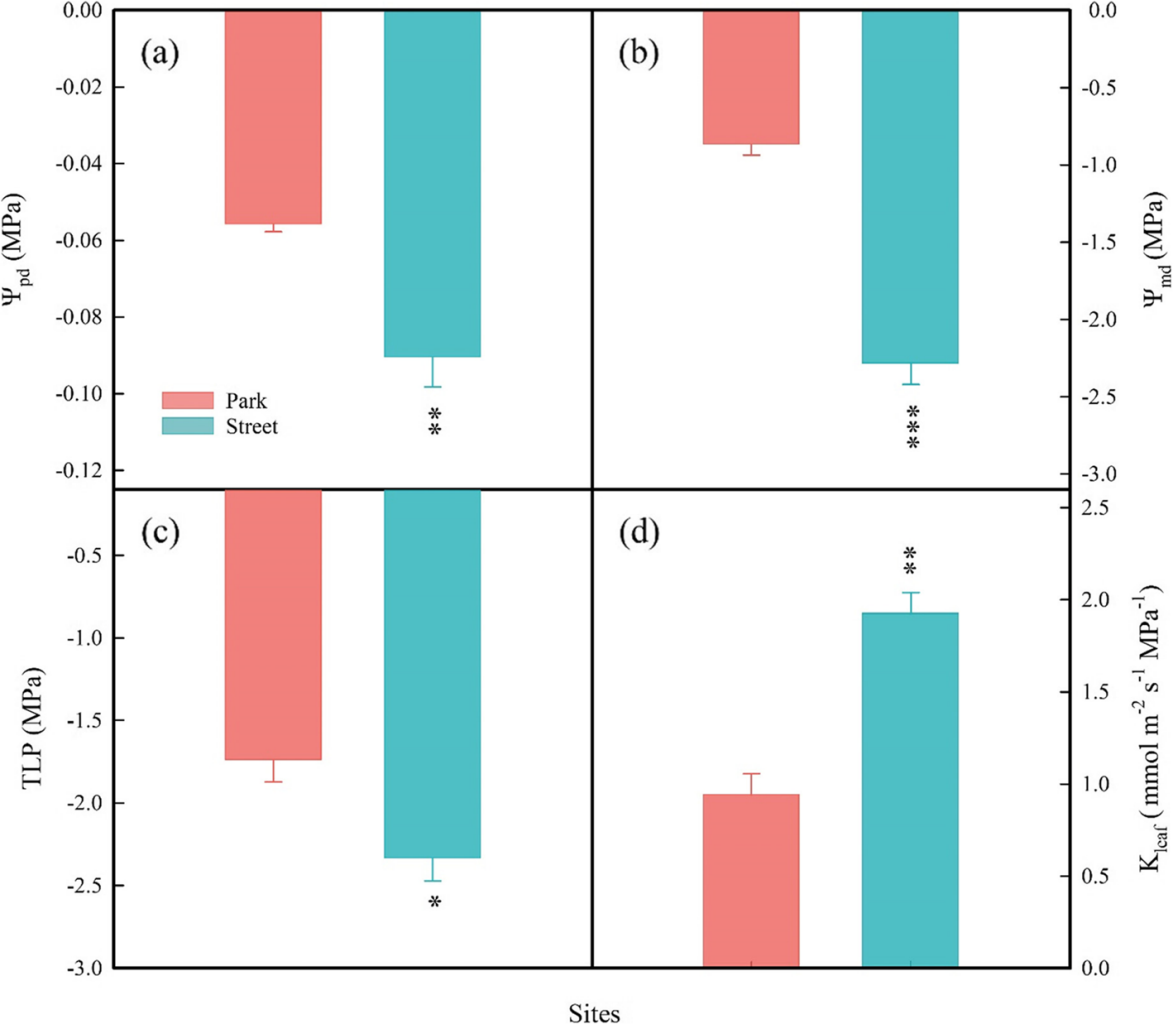
Predawn leaf water potential (Ψ_pd_; **a**), midday leaf water potential (Ψ_md_; **b**), leaf turgor loss point (TLP; **c**), and leaf hydraulic conductivity (K_leaf_; **d**) were measured on trees at study sites park (red) and street (blue). * *P* < 0.05; ** *P* < 0.01; *** *P* < 0.001

**Fig. 3:**
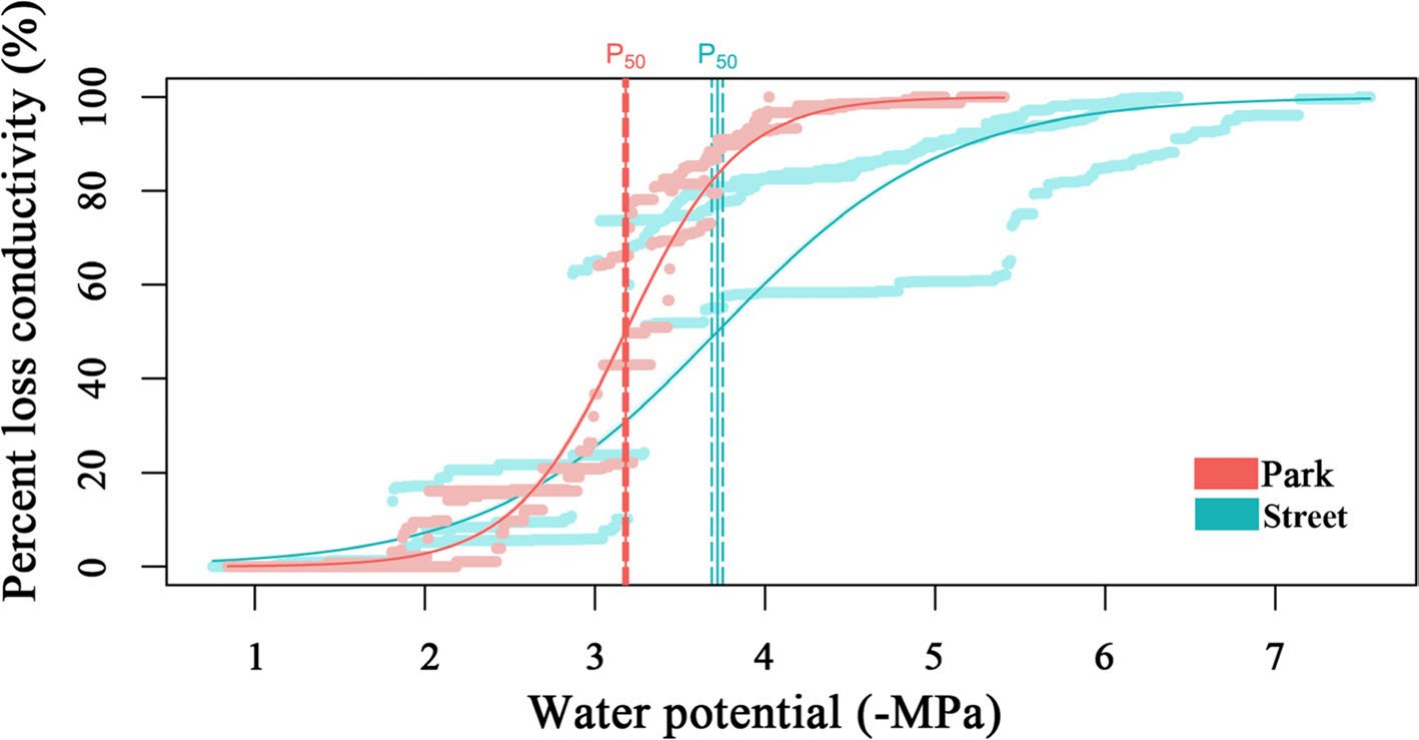
Percent loss of conductivity (PLC) as a function of xylem pressure for two study sites. Red symbols and line: park, blue symbols and line: street. Vertical dashed lines indicate the 95% confidence interval for P_50_ (estimated from the bootstrap). The shaded area represents 95% bootstrapped confidence interval for the fitted curve

**Fig. 4:**
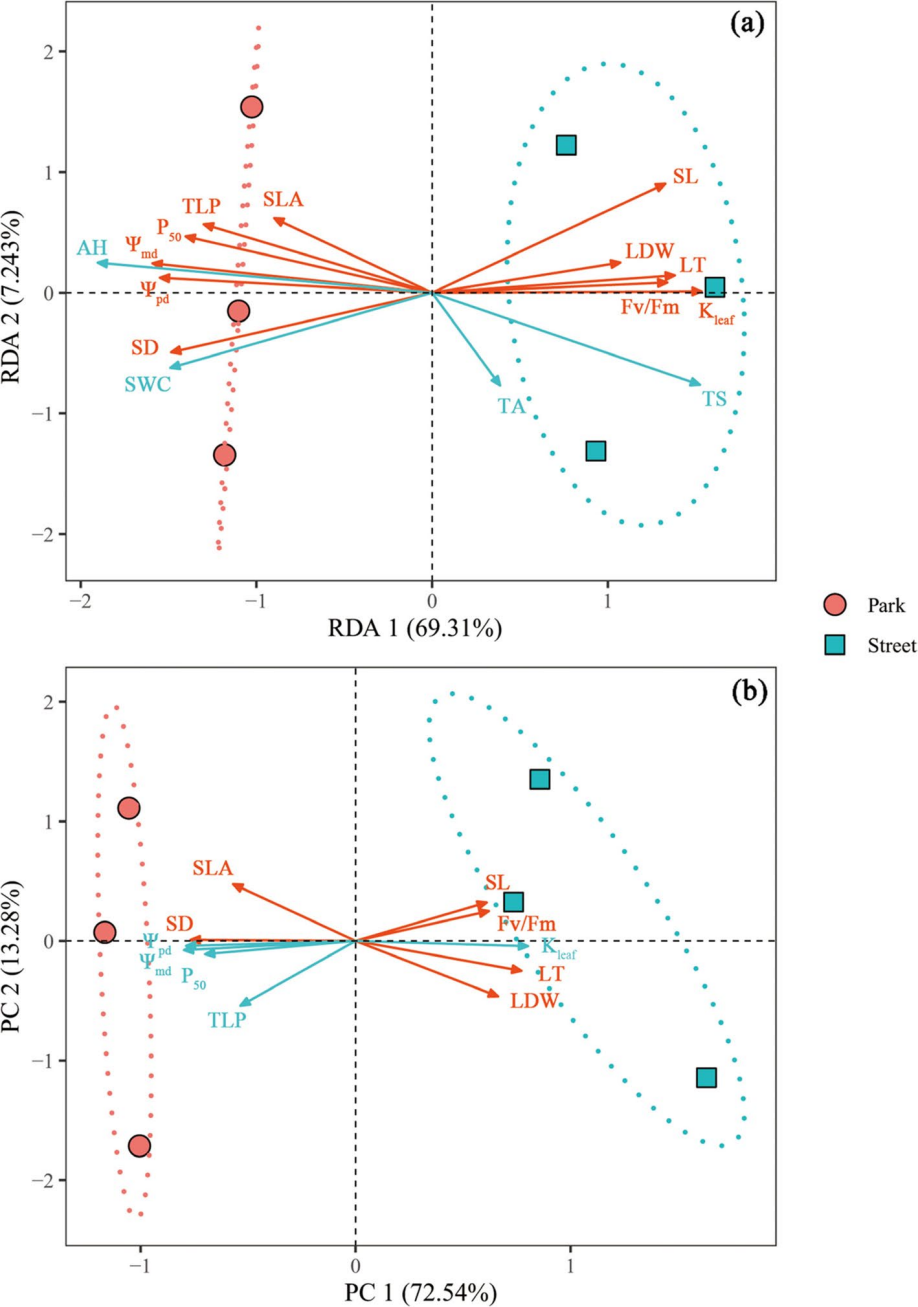
Analysis of multivariate associations of leaf functional traits and the relationship between environmental factors and leaf functional traits. **a** redundancy analysis (RDA) presenting leaf functional traits (red lines) and environmental factors (blue lines); **b** principal component analyses (PCA) on leaf hydraulic traits (blue lines) and economic traits (red lines). TA: air temperature, AH: air relative humidity, SWC: volumetric soil water content, TS: soil temperature, Ψ_pd_: predawn leaf water potential, Ψ_md_: midday leaf water potential, TLP: leaf turgor loss point, P_50_: leaf water potential at 50% loss of hydraulic conductance, K_leaf_: leaf hydraulic conductivity. LDW: leaf dry weight, LT: leaf thickness, SLA: specific leaf area, SD: stomatal density, SL: stomatal length, F_v_/F_m_: maximum photochemical quantum yield of photosystem II. Dashed ellipses indicate 95% confidence interval

Trees on the street had more negative predawn leaf water potential (Ψ_pd_; Fig. 2a), midday leaf water potential (Ψ_md_; Fig. 2b), leaf turgor loss point (TLP; Fig. 2c), and the leaf water potential at 50% loss of hydraulic conductance (P_50_; Fig. 3), but had higher leaf hydraulic conductivity (K_leaf_; Fig. 2d). The associations between leaf hydraulic traits and environmental factors from RDA were presented in Fig. 4a. According to Fig. 4a, the key factor affecting the Ψ_pd_, Ψ_md_, TLP, P_50_, and K_leaf_ is AH.

### Differences in leaf economic traits between street paved trees and park trees

**Fig. 5:**
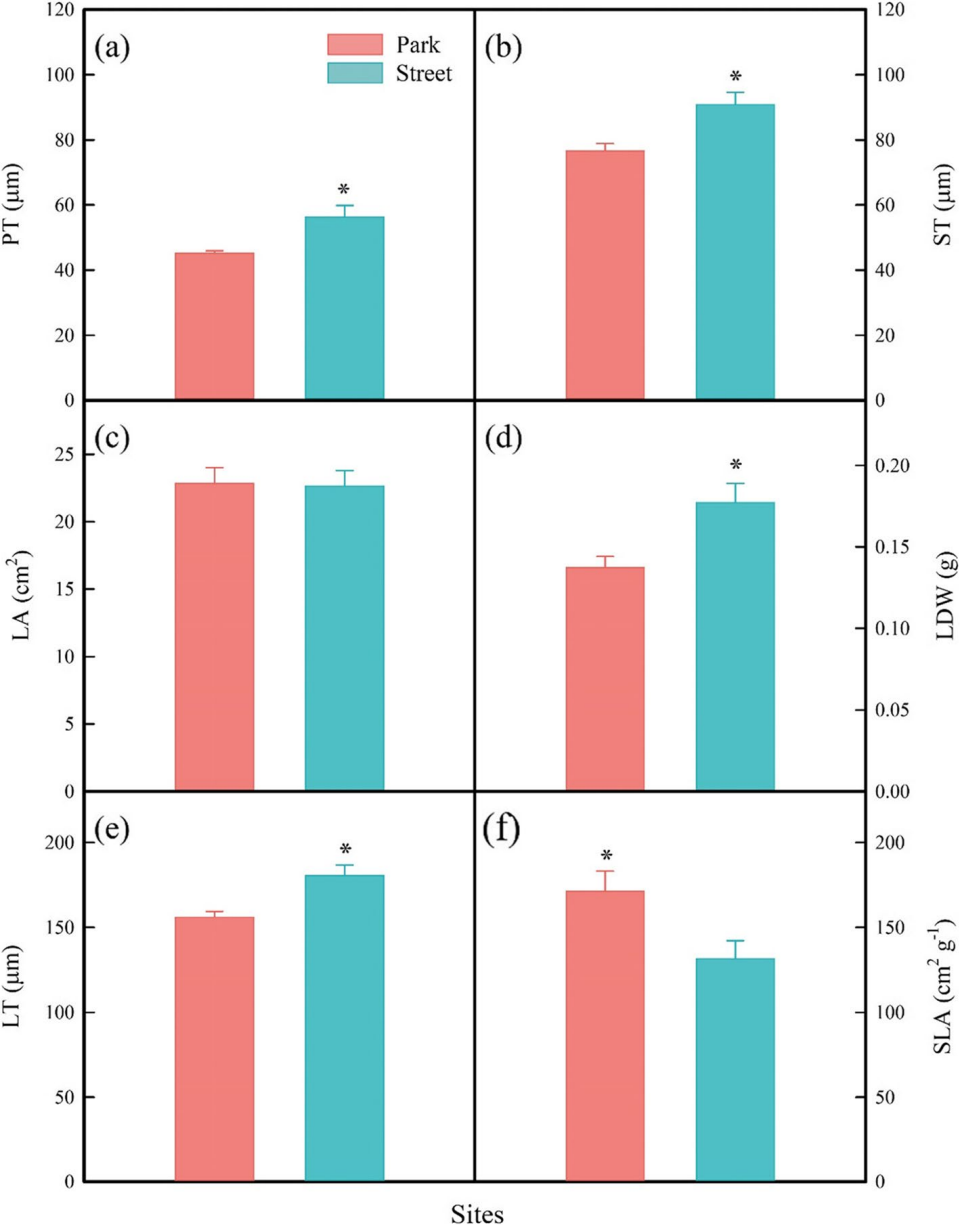
Palisade mesophyll thickness (PT; **a**), spongy mesophyll thickness (ST; **b**), leaf area (LA; **c**), leaf dry weight (LDW; **d**), leaf thickness (LT; **e**), and specific leaf area (SLA; **f**) were measured on trees at study sites park (red) and street (blue). * *P* < 0.05; ** *P* < 0.01; *** *P* < 0.001

**Fig. 6:**
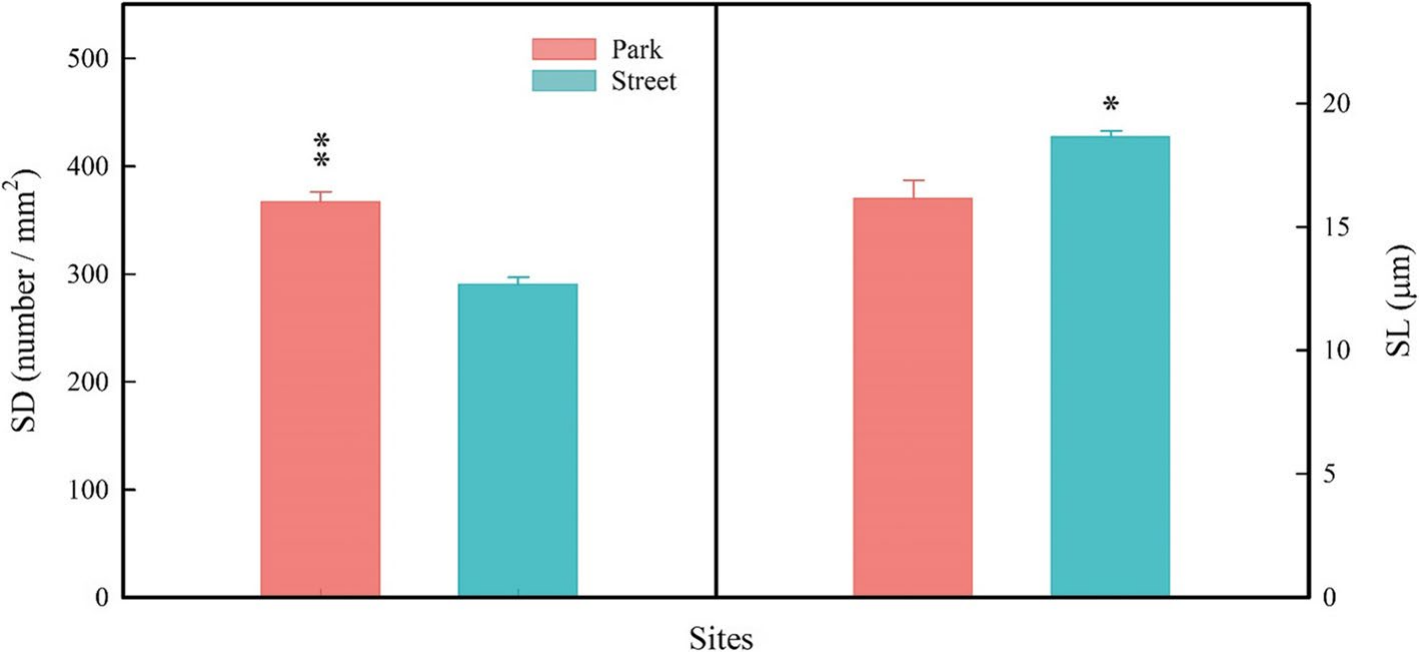
Stomatal density (SD; a) and stomatal length (SL; b) were measured on trees at study sites park (red) and street (blue). * *P* < 0.05; ** *P* < 0.01; *** *P* < 0.001

**Fig. 7:**
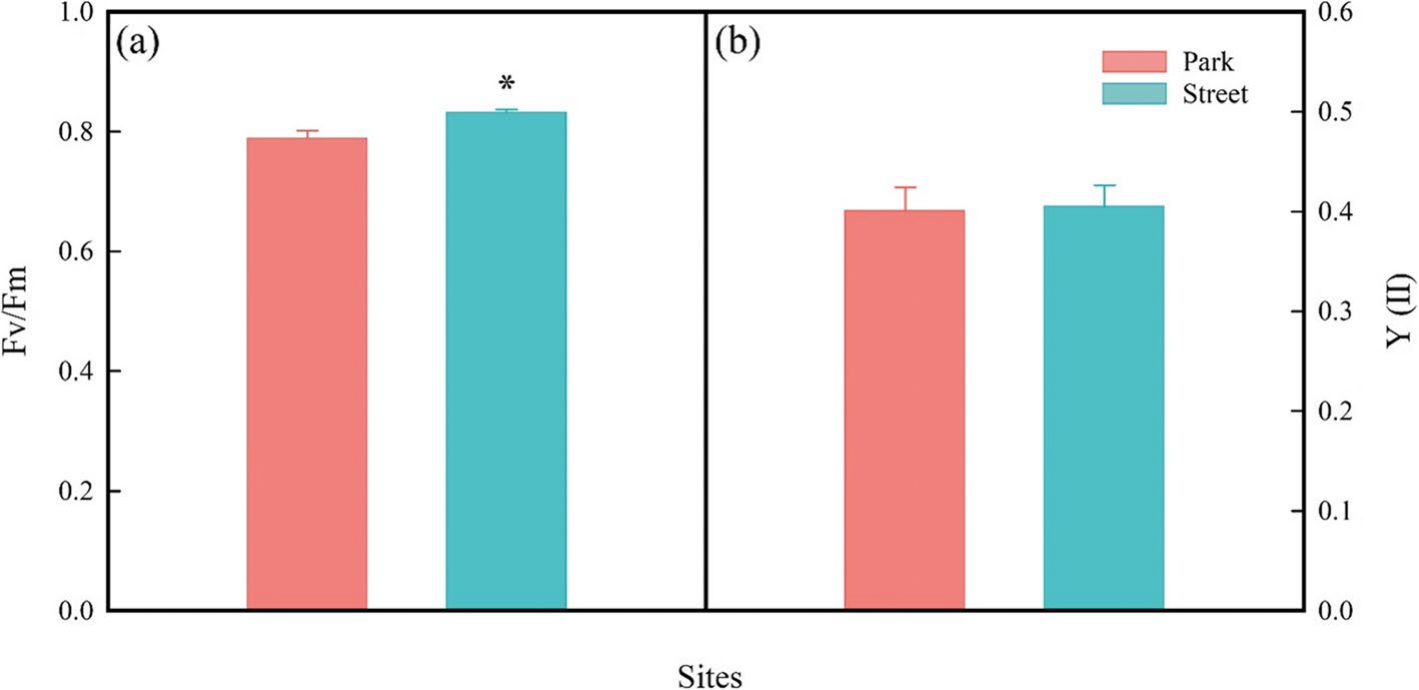
Maximum photochemical quantum yield of photosystem II (F_v_/F_m_; **a**) and photochemical quantum yield of photosystem II (Y(II); **b**) were measured on trees at study sites park (red) and street (blue). * *P* < 0.05; ** *P* < 0.01; *** *P* < 0.001

Leaf morphological traits were highly sensitive to the street pavement environment here. More specifically, the tree in the street has higher leaf dry weight (LDW; Fig. 5d) and higher leaf thickness (LT; Fig. 5e) but significantly lower SLA (Fig. 5f) than in the park. By contrast, there was no significant difference in leaf area (LA; Fig. 5c) of trees between the two sites. At the same time, the trees in the street pavement environment increased PT (Fig. 5a) and ST (Fig. 5b). In addition, the trees in the street pavement environment significantly reduces SD (Fig. 6a) while increasing SL (Fig. 6b). The fluorescence measurement of tree leaves growing in the park and the street is given in Fig. 7. There were significant differences between the F_v_/F_m_ in the two different sites, but the F_v_/F_m_ values are close to 0.8 (Fig. 7a). However, the Y(II) of the trees in the two sites was no significant difference (Fig. 7b). The RDA was used to analyze the relationship between leaf functional traits and environmental factors, explaining 76.55% of the variation in leaf functional traits (Fig. 4a). According to the RDA graph, SD and SLA were most affected by SWC and AH. Moreover, SL, LT, LDW, and F_v_/F_m_ were correlated with TS but were negatively correlated with SWC and AH.

### Coordinate with leaf hydraulic and economic traits acclimation to the street pavement environment

**Fig. 8:**
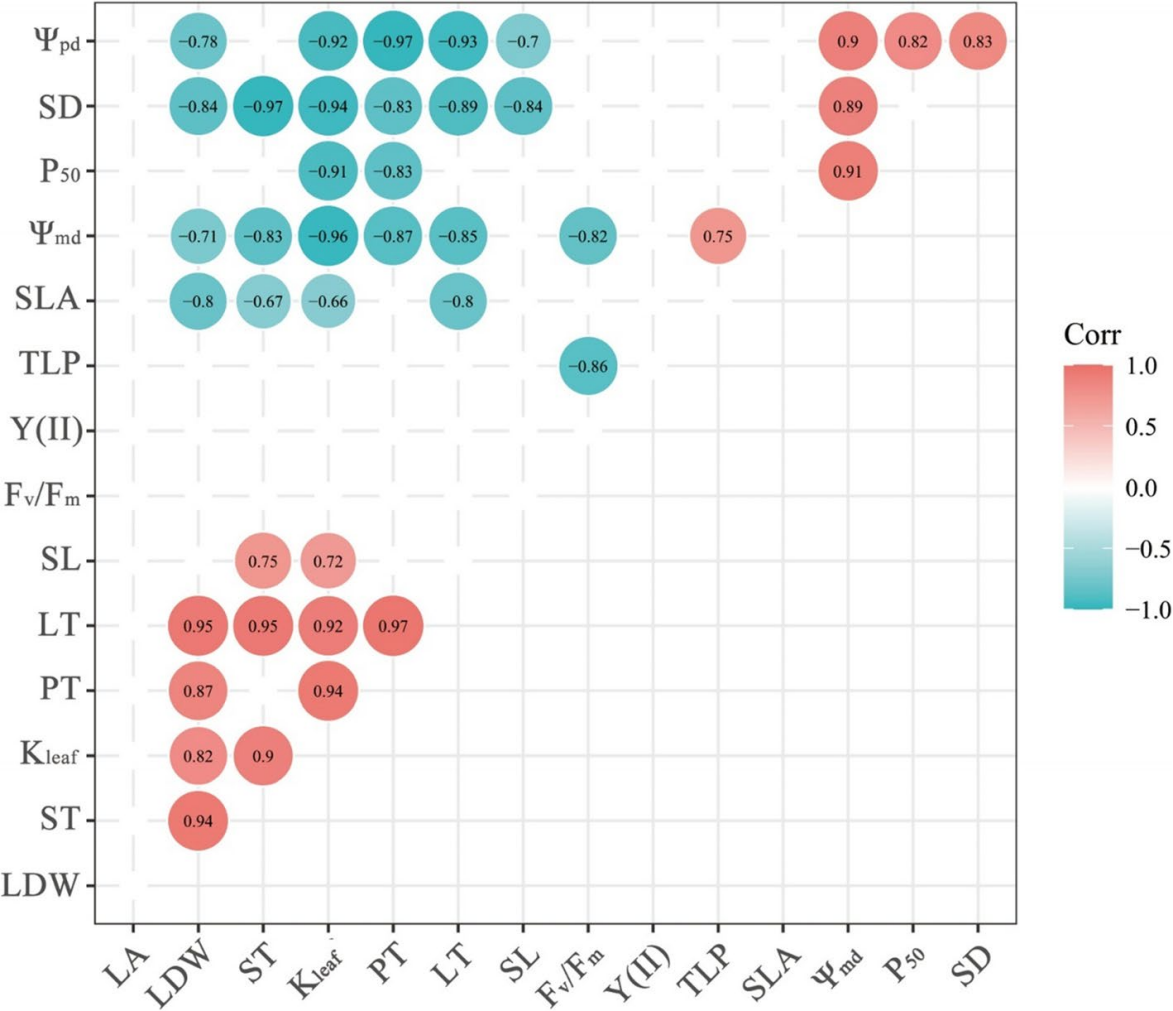
Heat map of correlation among plant functional traits. Ψ_pd_: predawn leaf water potential, Ψ_md_: midday leaf water potential, TLP: leaf turgor loss point, P_50_: leaf water potential at 50% loss of hydraulic conductance, K_leaf_: leaf hydraulic conductivity, PT: palisade mesophyll thickness, ST: spongy mesophyll thickness, LA: leaf area, LDW: leaf dry weight, LT: leaf thickness, SLA: specific leaf area, SD: stomatal density, SL: stomatal length, F_v_/F_m_: maximum photochemical quantum yield of photosystem II. Numbers represent Pearson correlation coefficients and only the colored circles are significant correlations at P-level < 0.05 (red: positive significant correlation; blue: negative significant correlation; blank: not-significant)

To determine the association between leaf hydraulic and economic traits in the street pavement environment, we performed principal analysis (PCA) on the mean values of leaf functional traits of *C. camphora* at two sites (Fig. 4b). The first PCA axis (PC1) explained most of this variation (72.54%). K_leaf_, SL, F_v_/F_m_, LT and LDW were positively correlated with PC1, while Ψ_pd_, Ψ_md_, P_50_, TLP, SD and SLA were negatively correlated with PC1. Two groups of traits can be distinguished, related to hydraulic and economic traits. At the right end of PC1 are street species with thick leaves, negative P_50_ and TLP, and low SLA. At the left end of PC1 are park species with low dry weight and high water potential. Leaf traits are not independent of each other, so correlations between leaf economic and hydraulic traits were observed (Figs. 8, S2). K_leaf_ and LT were positively correlated with LDW. However, SLA, Ψ_pd_ and Ψ_md_ were negatively correlated. TLP was negatively correlated with F_v_/F_m_ (Fig. S2f).

## Discussion

### The differences in the temperature and moisture of the air and soil in street pavement environment

Not only does street pavement reduce stormwater infiltration and surface runoff, but it also affects soil compaction, affecting soil ventilation and permeability, which in turn affects soil temperature and humidity. Our study showed that the street pavement significantly increased soil temperature, which is consistent with previous research reports that the soil temperature under the pavement is higher than that under vegetation cover [11, 48]. In addition to this, lower volumetric soil water content under the pavement was observed, as expected [49]. According to previous studies by Wang X et al., pavement increased soil temperature and decreased soil water content, which decreased the photosynthetic rate of *Fraxinus chinensis* and *Acer truncatum* and accelerated leaf budburst of *Fraxinus chinensis* [11, 50].

Changes in air temperature and relative humidity are expected as low albedo pavement materials significantly increase the absorbed solar radiation, resulting in elevated surface temperatures and severe long-wave radiation intensity [51]. Previous studies have shown that the increase of pavement surface temperature significantly reduced the biomass of street trees by 27.1–40.7%, and decreased the height and base diameter increment [12, 52]. However, the changes of air temperature and relative humidity could not be observed in this study because the pavement material in our experiments may have a high albedo. Some studies have found that pavement materials with high albedo can alleviate the heat island effect [9, 53].

### Effects of street pavement environment on plant hydraulic and economical traits

Urban plants grown in paved environments suffered significantly higher water stress than urban plants grown in vegetated cover [16]. We found that Ψ_pd_ and Ψ_md_ were more negative in street sites. These differences may be due to reduced water supply. According to the redundancy analysis, AH and SWC was the most important factor affecting plant water potential (Fig. 4a). It has been reported that the impervious pavement surfaces may prevent or slow rainwater infiltration in the soil, thereby reducing the amount of water available to plants [8]. Cavitation is prone to occur when the water supply is insufficient and the xylem is under negative pressure, but resistance to cavitation varies with species-specific vulnerabilities. Previous studies have reported that some tree species are susceptible to cavitation, and they usually respond to moderate drought by adjusting for lower P_50_ [54]. Our study also found that *C. camphora* has more negative leaf P_50_ and TLP to adapt to the street pavement environment. According to the classic trade-off between hydraulic efficiency and cavitation safety, species with higher safety tend to have lower hydraulic conductivity [55]. However, we found that trees in the street pavement environment have high leaf hydraulic conductivity (Fig. 2d). Leaf hydraulics are more complex because there are two main hydraulic pathways, the vein xylem and the extraxylem mesophyll pathway. The extraxylem pathway is the primary constraint on K_leaf_ [56, 57]. Therefore, the leaf water conduction mechanism of urban trees needs further research.

Leaf economic traits vary widely between the street and the park site. The street trees had higher LT, LDW, and SL and lower SLA and SD than those in the park (Figs. 5, 6). Trees with higher leaf thickness (LT) had the more substantial photosynthetic capacity and photodamage resistance by increasing the nitrogen content per unit leaf area and the volume of the photosynthetic machinery [58], which may explain the higher leaf thickness of trees at the street site in our study. And trees with lower SLA indicate a higher construction cost per unit area and increased light-harvesting potential, which is consistent with our findings that trees at the street site have higher F_v_/F_m_. Furthermore, plants with lower SLA tend to devote most of their energy and nutrients to building defensive structures or increasing leaf tissue density to prevent excessive dehydration [59]. In our study, the street trees may reduce the impact of a water-deficient environment and prevent excessive dehydration by reducing the specific leaf area and increasing the leaf thickness. Stomatal development, including stomatal size and density, is considered to be closely related to the adaptation mechanism of plants to environmental factors including temperature, air humidity, soil moisture content, CO_2_ concentration and vapor pressure deficit [20, 60]. As previously reported, leaf stomatal density decreased under the urban pavement environment in the present study. Lower stomatal density will reduce the water loss of plant leaves in a water-deficit environment to maintain water balance. It may be that the water-deficient environment of street pavement limits stomata density and increases the stomatal length to match the balance between water loss and carbon fixation capacity. Therefore, trees in the street increased stomatal length, which might be due to the decrease of stomatal density.

### Coordinate with leaf hydraulic and economic traits acclimation to street pavement environment

Yin et al. reported that the coupling of economic and hydraulic traits in leaves on the Chinese Loess Plateau depends on water availability [28]. We also found the coupling of leaf economic and hydraulic traits under the stress of the street pavement environment. Plants adapt to changing external environments and meet actual physiological needs by coordinating changes in their functional traits [61]. Plants with lower stomatal density response to water loss can adapt to fluctuating environmental conditions [25]. We detected that street trees have lower stomatal density than the park and may have higher water use efficiency [61]. At the same time, to ensure the progress of photosynthesis, plants will increase the stomatal length to ensure stomatal exchange and increase the thickness of the palisade tissue to improve the investment in the structure of photosynthetic tissue. We found that trees in the street pavement environment showed a slow investment-return type in the leaf economics spectrum, with lower specific leaf area, lower stomatal density, higher leaf thickness, higher leaf dry weight, and higher stomatal length. On the other hand, an efficient and safe water supply through leaf veins is essential for plant growth and survival, as leaves need to constantly replenish water lost due to stomata (transpiration) [62]. Species with a conservative strategy that is highly resistant to the embolism of its conducting system are more common in drier climates [30]. Our study showed that trees in the street pavement environment were more resistant to embolism than in the park, with more negative TLP and P_50_.

Taking the results of the above discussion together, the higher leaf hydraulic resistance is coordinated with the slower leaf economic strategy, ensuring the survival of the trees in the street pavement environment. Our PCA results further strengthened the coordination (Fig. 4b): the trees tested on the two sites were distributed along the PC 1, implying that leaf economic and hydraulic traits together determine the adaptation strategies of trees to the street pavement environment. Plant functional traits, as bridges connecting plants and the environment, play an essential role in studying how plants adapt to changing environments [63]. In conclusion, urban street trees form the best survival strategy to adapt to the street pavement environment through the adjustment and trade-off of leaf functional traits.

## Conclusion

This study compared the differences of leaf economic and hydraulic traits in a street site and a park site. The results showed that trees in the paved environment with low SWC and AH had more negative P_50_, lower specific leaf area (SLA), lower stomatal density (SD), longer stomatal length (SL), higher leaf dry weight (LDW) and thicker leaves compared to the park trees. Water availability may be a key factor contributing to the difference in leaf economics and hydraulic traits between the two sites. Under the influence of street pavement, *C. camphora* can improve its own drought resistance by increasing investment in leaf structure. *C. camphora* in the street exhibited a ‘slow investment return’ type on the leaf economic spectrum and high embolism resistance on the hydraulics. This indicated that *C. camphora* can coordinate leaf economic and hydraulic traits to adapt to the street pavement environment. It provided a theoretical basis for monitoring and predicting the impact of future urban paving environment changes on the physiological ecology of street trees. In the future, it is necessary to select several street trees and different pavement materials to further determine the impact of water availability on the coupling of economic and hydraulic traits in the pavement environment.

## Supplementary Information

**Additional file 1: Figure S1.** Monthly average air temperature (dots) and rainfall (bars) during 2010–2016 in Hefei. **Figure S2.** Scatter plot with a linear regression line showing correlation between leaf function traits. Red dots (park), blue dots(street). Ψ_md_: leaf midday water potential, TLP: leaf turgor loss point, P_50_: leaf water potential at 50% loss of hydraulic conductance, K_leaf_: leaf hydraulic conductivity, PT: palisade mesophyll thickness, LT: leaf thickness, F_v_/F_m_: maximum photochemical quantum yield of photosystem II.

## Data Availability

Datasets are available from the corresponding author on reasonable request.

## Abbreviations

TS: Soil temperature
SWC: Soil volumetric water content
TA: Air temperature
AH: Air relative humidity
Ψ_pd_: Predawn leaf water potential
Ψ_md_: Midday leaf water potential
TLP: Leaf turgor loss point
K_leaf_: Leaf hydraulic conductivity
P_50_: Leaf water potential at 50% loss of hydraulic conductance
PT: Palisade mesophyll thickness
ST: Spongy mesophyll thickness
LA: Leaf area
LDW: Leaf dry weight
LT: Leaf thickness
SLA: Specific leaf area
SD: Stomatal density
SL: Stomatal length
F_v_/F_m_: Maximum photochemical quantum yield of photosystem II
Y(II): Photochemical quantum yield of photosystem II
