# Supplementary material for: Coordination of leaf hydraulic and economic traits in Cinnamomum camphora under impervious pavement
Source: BMC Plant Biol. 2022 Jul 16;22:347. doi: 10.1186/s12870-022-03740-4 (PMC9287966; doi:10.1186/s12870-022-03740-4)

**Figure S1** Monthly average air temperature (dots) and rainfall (bars) during 2010-2016 in Hefei.


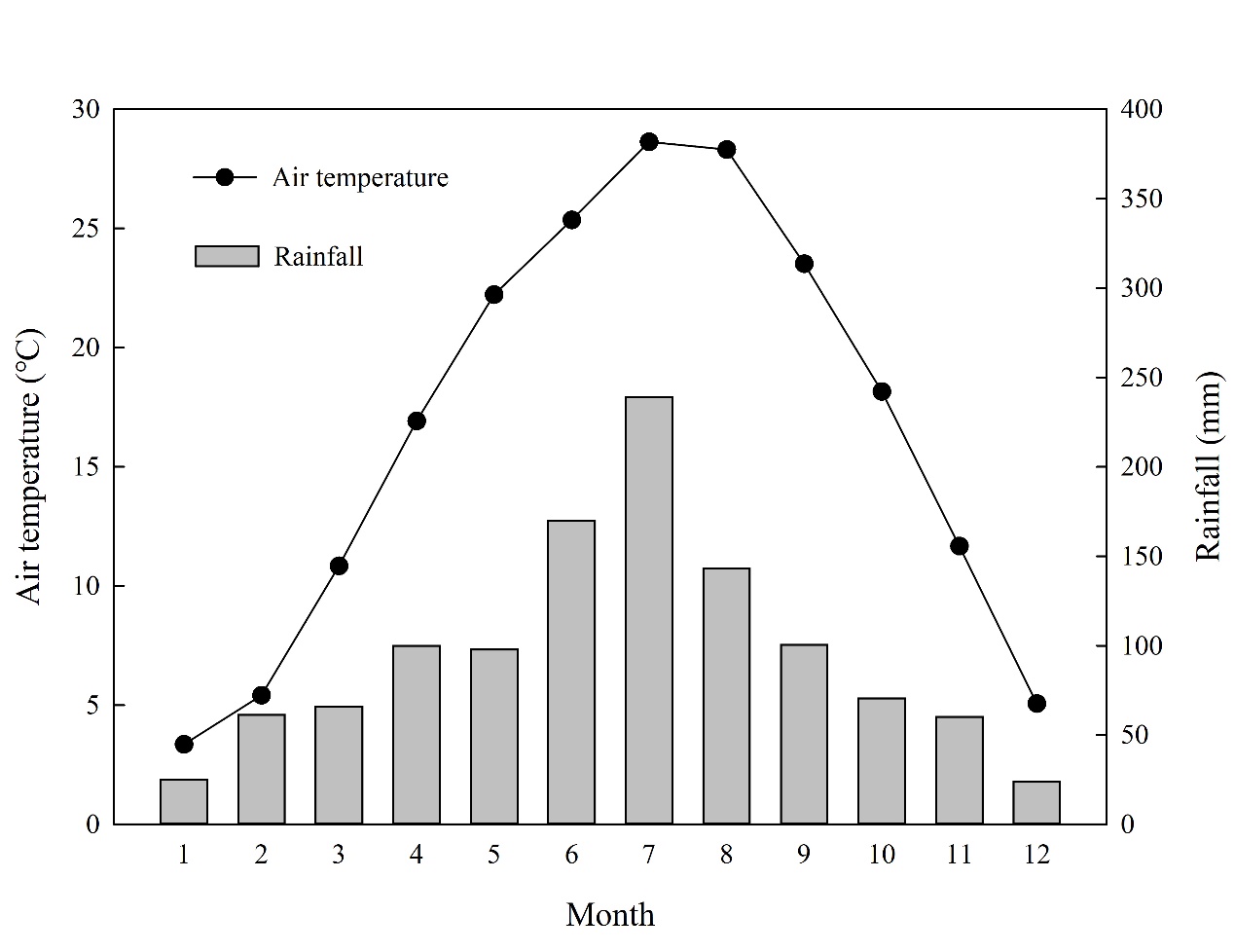


**Figure S2** Scatter plot with a linear regression line showing correlation between leaf function traits. Red dots (park), blue dots(street). Ψ_md_: leaf midday water potential, TLP: leaf turgor loss point, P_50_: leaf water potential at 50% loss of hydraulic conductance, K_leaf_: leaf hydraulic conductivity, PT: palisade mesophyll thickness, LT: leaf thickness, F_v_/F_m_: maximum photochemical quantum yield of photosystem II.


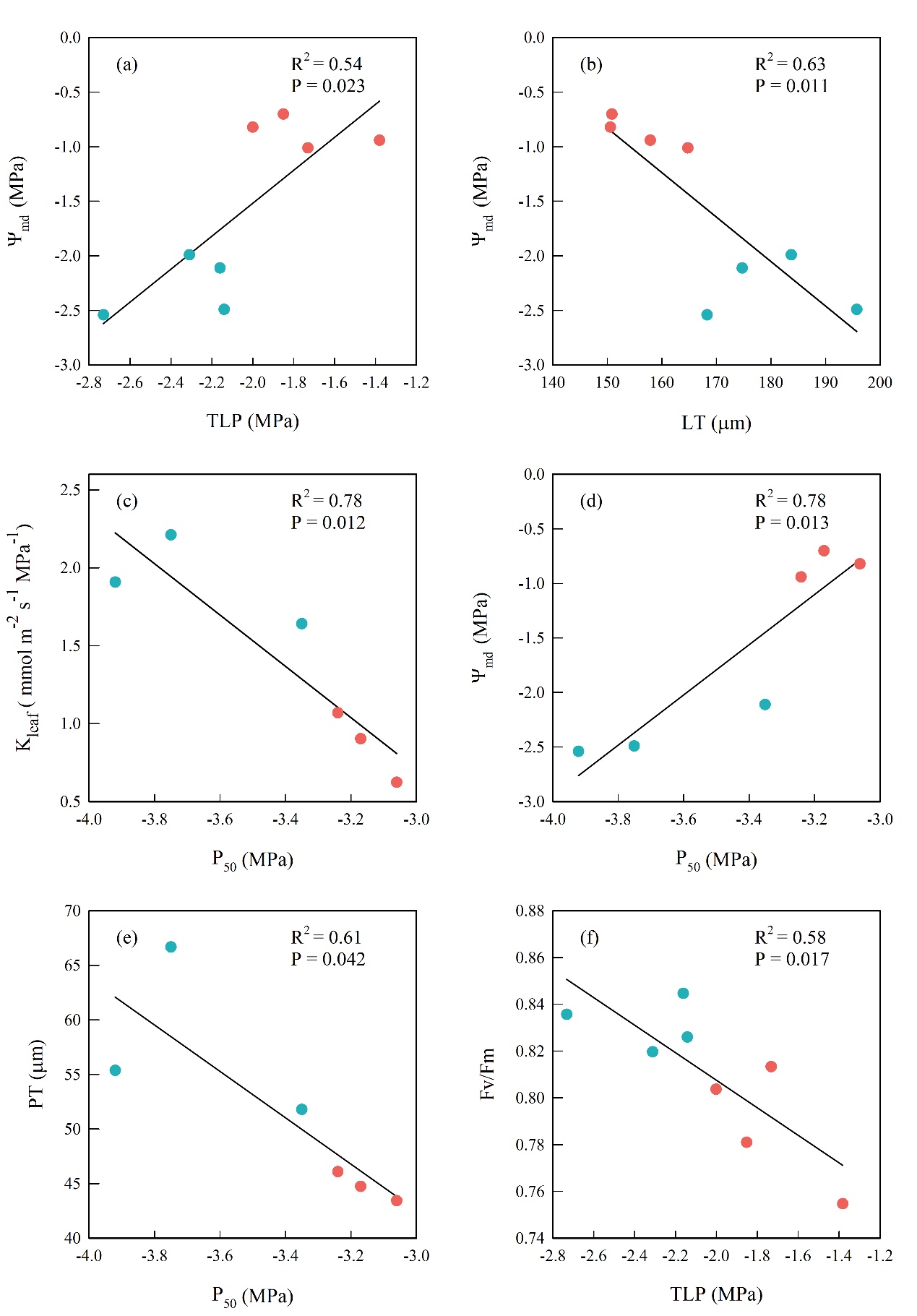

Supplement: Supplementary file 1 — Additional file 1: Figure S1. Monthly average air temperature (dots) and rainfall (bars) during 2010–2016 in Hefei. Figure S2. Scatter plot with a linear regression line showing correlation between leaf function traits. Red dots (park), blue dots(street). Ψmd: leaf midday water potential, TLP: leaf turgor loss point, P50: leaf water potential at 50% loss of hydraulic conductance, Kleaf: leaf hydraulic conductivity, PT: palisade mesophyll thickness, LT: leaf thickness, Fv/Fm: maximum photochemical quantum yield of photosystem II. [file 12870_2022_3740_MOESM1_ESM.docx]
